# Association Between the COVID-19 Pandemic and Disparities in Access to Major Surgery in the US

**DOI:** 10.1001/jamanetworkopen.2022.13527

**Published:** 2022-05-23

**Authors:** Laurent G. Glance, Eeshwar K. Chandrasekar, Ernie Shippey, Patricia W. Stone, Richard Dutton, Patrick J. McCormick, Jingjing Shang, Stewart J. Lustik, Isaac Y. Wu, Michael P. Eaton, Andrew W. Dick

**Affiliations:** 1 Department of Anesthesiology and Perioperative Medicine, University of Rochester School of Medicine, Rochester, New York; 2 Department of Public Health Sciences, University of Rochester School of Medicine, Rochester, New York; 3 RAND Health, RAND, Boston, Massachusetts; 4 Vizient Center for Advanced Analytics, Chicago, Illinois; 5 Columbia School of Nursing, Center for Health Policy, New York, New York; 6 US Anesthesia Partners, Dallas, Texas; 7 Memorial Sloan-Kettering Cancer Center, New York, New York

## Abstract

**Importance:**

Racial minority groups account for 70% of excess deaths not related to COVID-19. Understanding the association of the Centers for Medicare & Medicaid Services’ (CMS’s) moratorium delaying nonessential operations with racial disparities will help shape future pandemic responses.

**Objective:**

To evaluate the association of the CMS’s moratorium on elective operations during the first wave of the COVID-19 pandemic among Black individuals, Asian individuals, and individuals of other races compared with White individuals.

**Design, Setting, and Participants:**

This cross-sectional study assessed a 719-hospital retrospective cohort of 3 470 905 adult inpatient hospitalizations for major surgery between January 1, 2018, and October 31, 2020.

**Exposure:**

The first wave of COVID-19 infections between March 1, 2020, and May 31, 2020.

**Main Outcomes and Measures:**

The main outcome was the association between changes in monthly elective surgical case volumes and the first wave of COVID-19 infections as a function of patient race, evaluated using negative binomial regression analysis.

**Results:**

Among 3 470 905 adults (1 823 816 female [52.5%]) with inpatient hospitalizations for major surgery, 70 752 (2.0%) were Asian, 453 428 (13.1%) were Black, 2 696 929 (77.7%) were White, and 249 796 (7.2%) were individuals of other races. The number of monthly elective cases during the first wave was 49% (incident rate ratio [IRR], 0.49; 95% CI, 0.486-0.492; *P* < .001) compared with the baseline period. The relative reduction in unadjusted elective surgery cases for Black (unadjusted IRR, 0.99; 95% CI, 0.97-1.01; *P* = .36), Asian (unadjusted IRR, 1.08; 95% CI, 1.03-1.14; *P* = .001), and other race individuals (unadjusted IRR, 0.97; 95% CI, 0.95-1.00; *P* = .05) during the surge period compared with the baseline period was very close to the change in cases for White individuals. After adjustment for age, sex, comorbidities, and surgical procedure, there was still no evidence that the first wave of the pandemic was associated with disparities in access to elective surgery.

**Conclusions and Relevance:**

In this cross-sectional study, the CMS’s moratorium on nonessential operations was associated with a 51% reduction in elective operations. It was not associated with greater reductions in operations for racial minority individuals than for White individuals. This evidence suggests that the early response to the pandemic did not increase disparities in access to surgical care.

## Key Points

**Question:**

What is the association between the Centers for Medicare & Medicaid Services moratorium on elective operations during the first wave of the COVID-19 pandemic and changes in the monthly elective surgical case volumes among Black individuals, Asian individuals, and individuals of other races compared with White individuals?

**Findings:**

In this cross-sectional study of 3 470 905 adults undergoing major surgery, the reduction in elective surgery case volumes during the Centers for Medicare & Medicaid Services moratorium was not greater for Black individuals, Asian individuals, and individuals of other races than for White individuals.

**Meaning:**

These findings suggest that the early response to the pandemic did not increase disparities in access to surgical care.

## Teaser

This cross-sectional study of adults undergoing major surgery evaluates whether racial disparities in access to surgical care occurred during the first wave of the COVID-19 pandemic.

## Introduction

With more than 153 million cases and 3.2 million deaths as of May 2021, the COVID-19 pandemic has been one of the most significant public health challenges of the 21st century.^1^ As of May 2021, the US experienced more than 766 000 deaths attributable to COVID-19, with per capita mortality higher than most countries with a developed economy.^2,3^ This number is striking given that the US spends nearly 50% more of its gross domestic product on health care compared with the next highest-spending nation in the group of high-income countries.^4^ People of color have been disproportionately affected by COVID-19. Racial minorities account for 36% of COVID-19 deaths, 70% of excess deaths not related to COVID-19, and 58% of non–COVID-19 excess life-years lost.^5^ Some of this excess mortality may be attributable to difficulty obtaining medical and surgical care during the pandemic.^5^

On the basis of observations from other countries and recommendations from medical organizations, such as the American College of Surgeons, the Centers for Medicare & Medicaid Services (CMS) placed a moratorium on nonessential operations during the first wave of the pandemic.^6,7^ The moratorium was created to increase hospital capacity for the surge of patients with COVID-19 who required medical attention.^8^ In the Veterans Administration hospital system, there was a 91% reduction in elective surgery during the first wave. Other hospital systems experienced similar decreases.^9^ Although the moratorium increased hospitals’ capacity to care for patients with COVID-19, it may have also led to significant delays in surgical care, contributing to excess non-COVID deaths. In the US, non-White individuals have historically had less access to essential medical and surgical care than White individuals.^10^ Although COVID-19 has led to similar decreases in medical admissions across demographic subgroups,^11^ it is unknown whether the moratorium on nonessential surgery disproportionately affected racial minority individuals compared with White individuals.

To better understand potential disparities in surgical access caused by COVID-19, we explored the association of the first wave of the pandemic on surgical case volumes among Black individuals, Asian individuals, and individuals of other minority races using data from more than 95% of academic medical centers and their affiliated hospitals in the US.^12^ We hypothesized that racial minority individuals would experience greater reductions in elective operations compared with White individuals. We separately examined elective, urgent, and emergency operations. We also performed analyses in patients undergoing time-sensitive surgical procedures (cardiac, thoracic, and colorectal) for whom surgical delays could lead to excess mortality and in patients undergoing elective hip and knee replacements for whom delays would not be expected to lead to excess mortality. Characterizing the association of the CMS’s moratorium on elective operations during the first wave of the COVID-19 pandemic on racial minority groups will help policy makers better respond to future events that stress our health care system while decreasing inequities.

## Methods

### Data Source

This cross-sectional study was conducted using data from the Vizient Clinical Database, formerly known as the University HealthSystem Consortium, which includes data on patients hospitalized at most academic medical centers and their affiliated hospitals in 50 states.^13^ This database includes information on patient demographic characteristics (age, sex, and self-reported race and ethnicity), admission status (elective, urgent, or emergency), and *International Statistical Classification of Diseases and Related Health Problems, Tenth Revision* diagnosis and procedure codes. The University of Rochester Research Study Review Board reviewed this study and determined that it met federal and university criteria for exemption because it consisted of secondary research on existing data. The statistical analysis plan for this study was filed with and reviewed by the University of Rochester Research Study Review Board before undertaking data cleaning and analysis. This study followed the Strengthening the Reporting of Observational Studies in Epidemiology (STROBE) reporting guideline.^14^

### Study Population

We identified 3 470 905 adult inpatient hospitalizations between January 1, 2018, and October 31, 2020, for major surgery at 719 hospitals. We grouped operations using the coding algorithm from the Centers for Disease Control and Prevention National Healthcare Safety Network.^15^ We included the following major operations in the analysis: abdominal aortic aneurysm repair; limb amputations; appendix surgery; bile duct, liver, or pancreatic surgery; coronary artery bypass graft (CABG) surgery; cardiac surgery (eg, aortic valve replacement); carotid endarterectomy; cholecystectomy; colon surgery; craniotomy; spinal fusion; fracture surgery; gastric surgery; knee arthroplasty; hip arthroplasty; laminectomy; peripheral vascular bypass surgery; small-bowel surgery; thoracic surgery; and exploratory laparotomy. We excluded patients with missing information on admission status (n = 3077), age (n = 5), sex (n = 257), and race (n = 71 930). The final analytic data set consisted of 3 470 905 patients (eFigure 1 in the Supplement).

### Outcome and Exposure

The primary outcome of interest was the number of major operations. The exposure of interest was the first wave (ie, surge) of the COVID-19 pandemic, defined as March 1, 2020, to May 31, 2020. We defined the baseline period as January 1, 2018, to February 29, 2020, and the postsurge period as June 1, 2020, to October 31, 2020. Our goal was to examine the differential association of the pandemic on individuals of Asian, Black, White, and other races. All individuals of races other than Asian, Black, and White were grouped in the other category because the original data were categorized in this manner. We did not use Hispanic ethnicity in our analysis because of the large amount of missing data (ie, the number of patients with missing ethnicity was similar to the number of patients with Hispanic ethnicity).

### Statistical Analysis

We used negative binomial regression to estimate the unadjusted monthly elective surgical case volumes as a function of race during the surge and postsurge periods by specifying the period and race as main effects and an interaction between period and race: *f*[*E*(*Y_tr_*) = β_0_ + β*_1t_Time* + β_2r_*Race* + β*_3tr_Time_t_xRace_r_* (model 1), where *f* is the log link function, *Y_tr_* is the monthly count of operations during the study period *Time* (surge and postsurge periods, with baseline period as the referent) for patient race specified with *Race* (Asian, Black, or other race, with White as the referent). We grouped patients with the same characteristics into separate bins (eg, Black patients undergoing surgery during the baseline period constitute a single bin). The bins served as the unit of analysis. We used the log of the number of months in each period as the offset in the negative binomial regression model. We characterized the relative change in surgical case volume between the baseline and the surge and postsurge periods for all races compared with White individuals using β_3_*_tr_*.

We also estimated an equivalent model in which we specified a categorical variable that incorporated both race and the period: *f*[*E*(*Y_tr_*)] = β_0_ + β_1_*_tr_TimeRace* (model 2; eg, when *TimeRace* = 1, race = White, and period = baseline). Model 1 and model 2 are identical. That is, linear combinations of the estimates in model 1 yield the estimates in model 2. We characterized the case volumes for all races relative to White individuals during the baseline period using β_1_*_tr_*.

We then reestimated these models using negative binomial regression adjusting for age, sex, comorbidity count, and procedure category (based on the first *Current Procedural Terminology* code listed). We grouped patients with the same characteristics into separate bins (eg, Black female patients between the ages of 31 and 50 years with 1-2 comorbidities undergoing CABG surgery during the baseline period constitute a single bin). The bins served as the unit of analysis. We consider the results of the unconditional analyses (that did not adjust for patient factors) as the main analyses because these results represent actual changes in case volumes associated with the COVID-19 pandemic. The adjusted analyses account for differences in patient case mix and procedures that resulted from changes in decision-making by surgeons and patients during the pandemic.

Next, we separately estimated these models for urgent and emergency surgical case volumes. We also performed 3 sensitivity analyses limiting elective operations to (1) cardiac surgery and CABG surgery, (2) colorectal and thoracic surgery, and (3) hip and knee arthroplasties.

Data management and statistical analyses were performed using Stata SE/MP software, version 17.0 (StataCorp LLC). We used robust variance estimators in the adjusted analyses. All statistical tests were 2-tailed, and *P* < .005 was considered significant after adjusting for multiple comparisons using the Bonferroni method.

## Results

Among 3 470 905 adults (1 823 816 female [52.5%] and 1 647 089 male [47.5%]) with inpatient hospitalizations for major surgery, 70 752 (2.0%) were Asian, 453 428 (13.1%) were Black, 2 696 929 (77.7%) were White, and 249 796 (7.2%) were individuals of other races (Table). A total of 2 074 527 admissions (59.8%) were elective and 1 067 302 (30.8%) were emergency. The 3 most common surgical procedures were hip arthroplasty (390 262 [11.2%]), knee arthroplasty (378 972 [10.9%]), and spinal fusion (334 864 [9.7%]). Of the 719 hospitals, 114 (15.9%) had 500 or more beds, and 342 (47.6%) had fewer than 100 beds (eTable 1 in the Supplement). A total of 351 hospitals (48.8%) had a peak inpatient COVID-19 census less than 5%, and 84 (11.7%) had a peak inpatient COVID-19 census greater than 25%.

**Table.** Patient Characteristics

Characteristic	No. (%) of patients			
	Total (N = 3 470 905)	Baseline (Jan 2018 to Feb 2020) (n = 2 771 278)	Surge (Mar 2020 to May 2020) (n = 203 072)	Postsurge (Jun 2020 to Oct 2020) (n = 496 555)
Race				
White	2 696 929 (77.7)	2 160 619 (78.0)	156 101 (76.9)	380 209 (76.6)
Black	453 428 (13.1)	357 348 (12.9)	27 799 (13.7)	68 281 (13.8)
Asian	70 752 (2.0)	56 267 (2.0)	4250 (2.1)	10 235 (2.1)
Other^a^	249 796 (7.2)	197 044 (7.1)	14 922 (7.4)	37 830 (7.6)
Sex				
Male	1 647 089 (47.5)	1 309 603 (47.3)	101 357 (49.9)	236 129 (47.6)
Female	1 823 816 (52.5)	1 461 675 (52.7)	101 715 (50.1)	260 426 (52.5)
Age group, y				
18-30	179 789 (5.2)	139 715 (5.0)	11 658 (5.7)	28 416 (5.7)
31-50	619 656 (17.9)	489 003 (17.7)	37 243 (18.3)	93 410 (18.8)
51-64	1 076 539 (31)	864 005 (31.2)	61 721 (30.4)	150 813 (30.4)
65-74	927 971 (26.7)	746 179 (26.9)	52 145 (25.7)	129 647 (26.1)
75-79	321 066 (9.3)	257 750 (9.3)	18 237 (9.0)	45 079 (9.1)
80-84	190 352 (5.5)	152 182 (5.5)	11 209 (5.5)	26 961 (5.4)
85-89	101 539 (2.9)	80 549 (2.9)	6705 (3.3)	14 285 (2.9)
≥90	53 993 (1.6)	41 895 (1.5)	4154 (2.1)	7944 (1.6)
Comorbidity count				
None	1 360 557 (39.2)	1 110 850 (40.1)	68 875 (33.9)	180 832 (36.4)
1-2	1 599 987 (46.1)	1 271 545 (45.9)	95 368 (47.0)	233 074 (46.9)
≥3	510 361 (14.7)	388 883 (14.0)	38 829 (19.1)	82 649 (16.6)
Admission status				
Emergency	1 067 302 (30.8)	818 890 (29.6)	82 744 (40.8)	165 668 (33.4)
Urgent	329 076 (9.5)	256 300 (9.3)	24 626 (12.1)	48 150 (9.7)
Elective	2 074 527 (59.8)	1 696 088 (61.2)	95 702 (47.1)	282 737 (56.9)
Procedure				
Abdominal aortic aneurysm	4235 (0.1)	3438 (0.1)	222 (0.1)	575 (0.1)
Limb amputation	158 664 (4.6)	121 101 (4.4)	12 932 (6.4)	24 631 (5.0)
Appendix surgery	93 633 (2.7)	72 664 (2.6)	6864 (3.4)	14 105 (2.8)
Bile duct surgery	104 180 (3.0)	80 492 (2.9)	7427 (3.7)	16 261 (3.3)
Liver or pancreatic surgery	154 948 (4.5)	123 272 (4.5)	9194 (4.5)	22 482 (4.5)
Cardiac surgery	110 902 (3.2)	88 862 (3.2)	6668 (3.3)	15 372 (3.1)
CABG	8479 (0.2)	6792 (0.3)	488 (0.2)	1199 (0.2)
Carotid endarterectomy	44 146 (1.3)	34 773 (1.3)	2747 (1.4)	6626 (1.3)
Cholecystectomy	183 931 (5.3)	144 442 (5.2)	12 344 (6.1)	27 145 (5.5)
Colon surgery	246 766 (7.1)	192 988 (7.0)	16 387 (8.1)	37 391 (7.5)
Craniotomy	275 639 (7.9)	213 124 (7.7)	19 817 (9.8)	42 698 (8.6)
Spinal fusion	334 864 (9.7)	265 209 (9.6)	17 781 (8.8)	51 874 (10.5)
Fracture surgery	212 776 (6.1)	163 517 (5.9)	16 440 (8.1)	32 819 (6.6)
Gastric surgery	164 167 (4.7)	131 024 (4.7)	7493 (3.7)	25 650 (5.2)
Knee arthroplasty	378 972 (10.9)	318 553 (11.5)	16 234 (8.0)	44 185 (8.9)
Hip arthroplasty	390 262 (11.2)	336 785 (12.2)	11 432 (5.6)	42 045 (8.5)
Laminectomy	80 421 (2.3)	64 831 (2.3)	4193 (2.1)	11 397 (2.3)
Peripheral vascular bypass surgery	46 008 (1.3)	35 739 (1.3)	3184 (1.6)	7085 (1.4)
Small-bowel surgery	147 610 (4.3)	114 417 (4.1)	8793 (4.3)	24 400 (4.9)
Thoracic surgery	154 662 (4.5)	122 019 (4.4)	10 659 (5.3)	21 984 (4.4)
Exploratory laparotomy	175 640 (5.1)	137 236 (5.0)	11 773 (5.8)	26 631 (5.4)

Abbreviation: CABG, coronary artery bypass graft surgery.

^a^ All individuals of races other than White, Black, and Asian were grouped in the other category because the original data were categorized in this manner.

**Figure 1.**
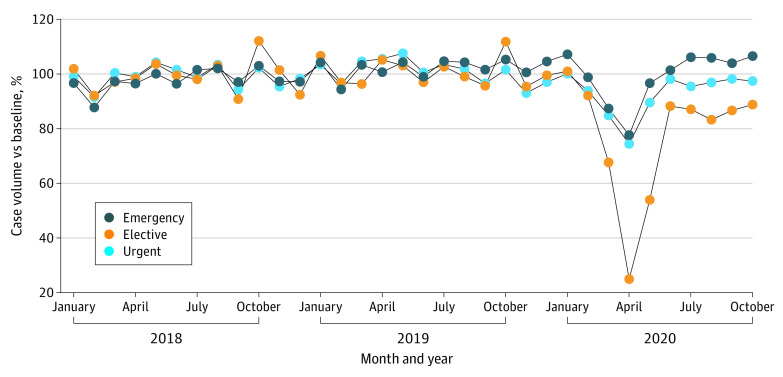
Surgery Case Volume Stratified by Urgency Monthly case volumes were normalized by dividing monthly case volume by mean case volume during the baseline period (January 1, 2018, to February 29, 2020).

The number of monthly elective cases during the surge period was 49% (incident rate ratio [IRR], 0.49; 95% CI, 0.486-0.492; *P* < .001) compared with the monthly number of cases in the baseline period, whereas the monthly number of urgent cases was 83% (IRR, 0.83; 95% CI, 0.82-0.84; *P* < .001) and the monthly number of emergency cases was 87.0% (IRR, 0.87; 95% CI, 0.87-0.88; *P* < .001) compared with the number of monthly cases (hereafter referred to as cases) in the baseline period (Figure 1; eFigure 2 in the Supplement). Although statistically significant, the number of cases during the postsurge period was close to the baseline for urgent (unadjusted IRR, 0.97; 95% CI, 0.96-0.98; *P* < .001) and emergency operations (unadjusted IRR, 1.05; 95% CI, 1.05-1.06; *P* < .001) but was still significantly less than the baseline for elective operations (unadjusted IRR, 0.87; 95% CI, 0.86-0.87; *P* < .001).

**Figure 2.**
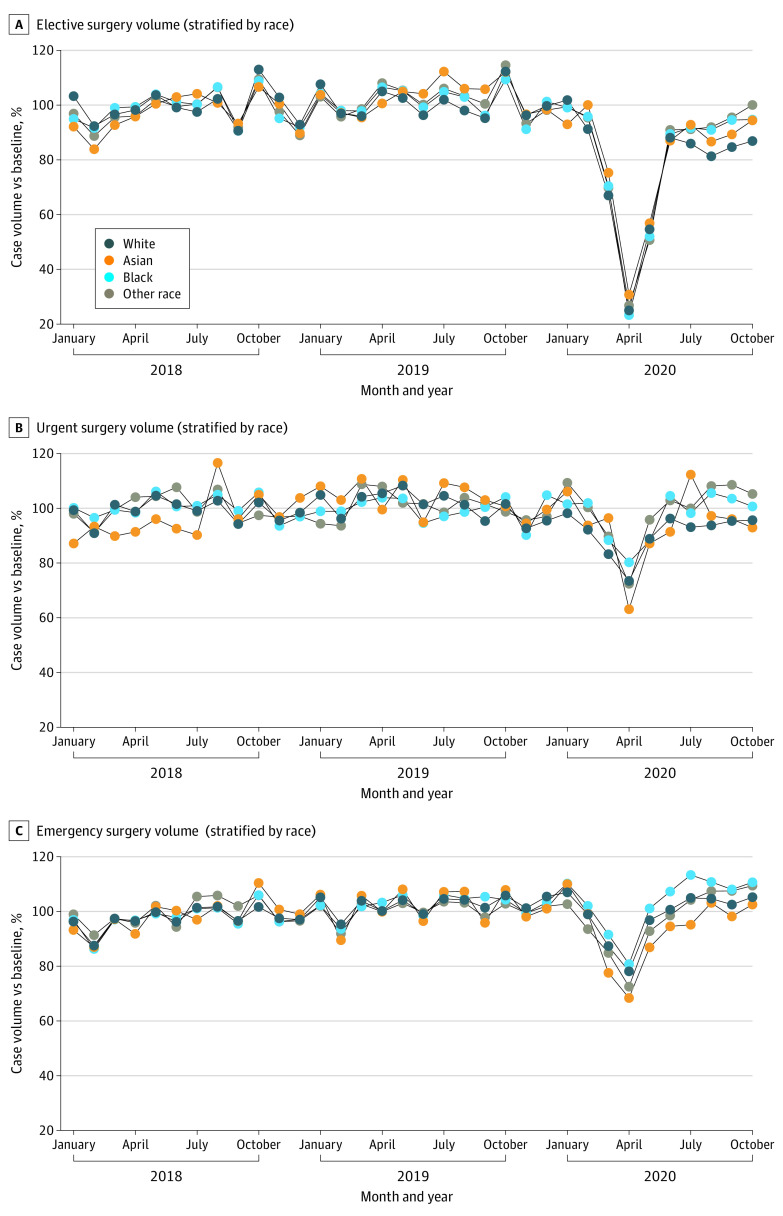
Surgical Case Volumes Stratified by Race and Urgency of Cases Monthly case volumes were normalized by dividing monthly case volume by mean case volume during the baseline period (January 1, 2018, to February 29, 2020).

**Figure 3.**
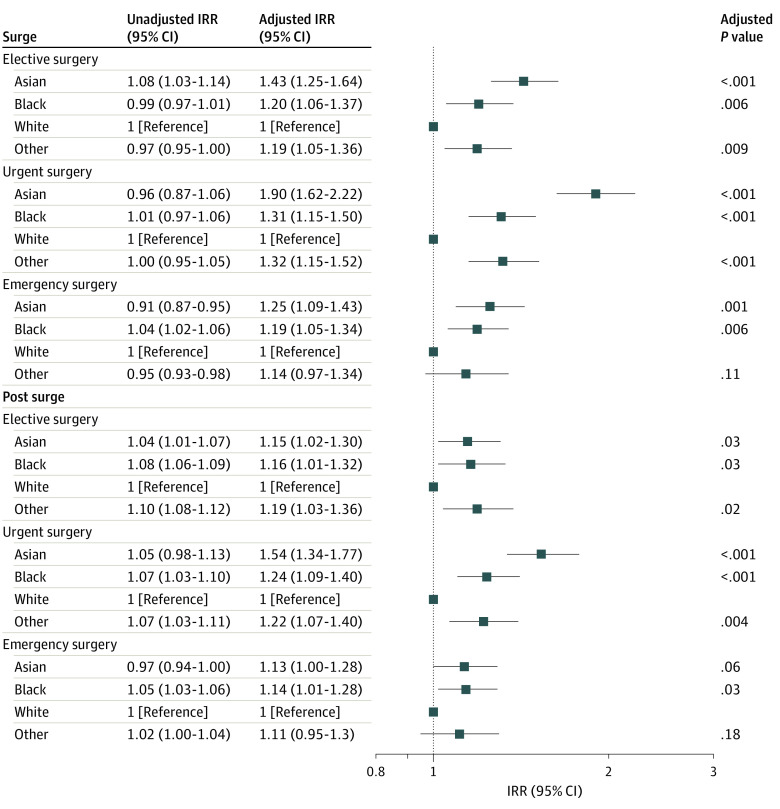
Changes in Surgical Case Volumes by Race Changes in surgical case volumes during the surge and postsurge periods compared with surgical case volumes for White patients during the baseline period (March 1, 2020, to May 31, 2020) stratified by race and surgical urgency. Results are based on model 2 in which race and period are specified as main effects. IRR indicates incident rate ratio.

eFigure 3 in the Supplement shows the ratio of surgical case volumes during the baseline, surge, and postsurge periods for individuals of Asian, Black, White, and other race compared with White patients during the baseline period. Compared with the baseline period, the unadjusted elective surgical case volumes for White patients were 49% (unadjusted IRR, 0.49; 95% CI, 0.49-0.49; *P* < .001) during the surge period and 85% (unadjusted IRR, 0.85; 95% CI, 0.85-0.86; *P* < .001) during the postsurge period (eTable 3 in the Supplement). Figure 2 and Figure 3 show the change in unadjusted case volumes during the surge and postsurge periods compared with the baseline period for Asian patients, Black patients, and patients of other race compared with White patients. These results are shown separately for elective, urgent, and emergency surgery. For elective surgery, the change in unadjusted surgery case volumes during the surge compared with the baseline period for each race relative to White patients was small: Asian, unadjusted IRR, 1.08; 95% CI, 1.03-1.14; *P* = .001; Black, unadjusted IRR, 0.99; 95% CI, 0.97-1.01; *P* = .36; and other race, unadjusted IRR, 0.97; 95% CI, 0.95-1.00; *P* = .05 (eTable 2 in the Supplement). Similarly, Black patients (unadjusted IRR, 1.08; 95% CI, 1.06-1.09; *P* < .001), Asian patients (unadjusted IRR, 1.04; 95% CI, 1.01-1.07; *P* = .007), and patients of other race (unadjusted IRR, 1.10; 95% CI, 1.08-1.12; *P* < .001) also experienced relative changes in elective surgical case volumes during the postsurge period compared with the baseline period that were similar to the changes in elective case volumes for White patients during the postsurge period (Figure 3).

Compared with the baseline period, the unadjusted emergency surgical case volumes for White patients were 88% (unadjusted IRR, 0.88; 95% CI, 0.87-0.88; *P* < .001) during the surge period and 104% (unadjusted IRR, 1.04; 95% CI, 1.04-1.05, *P* < .001) during the postsurge period (eFigure 3 and eTable 2 in the Supplement). For emergency surgery, the unadjusted ratio of surgical case volumes for Asian patients (unadjusted IRR, 0.91; 95% CI, 0.87-0.95; *P* < .001), Black patients (unadjusted IRR, 1.04; 95% CI, 1.02-1.06; *P* < .001), and patients of other race (unadjusted IRR, 0.95; 95% CI, 0.93-0.98; *P* < .001) during the surge period compared with the baseline period were close to the change in case volumes for White patients during the surge period compared with the baseline period (Figure 3; eTable 2 in the Supplement). Similarly, Asian patients (unadjusted IRR, 0.97; 95% CI, 0.94-1.00; *P* = .06), Black patients (unadjusted IRR, 1.05; 95% CI, 1.03-1.06; *P* < .001), and patients of other race (unadjusted IRR, 1.02; 95% CI, 1.00-1.04; *P* = .03) also experienced unadjusted changes in emergency surgical case volumes during the postsurge period that were similar to the changes in emergency case volumes for White patients during the postsurge period (Figure 3; eTable 2 in the Supplement).

**Figure 4.**
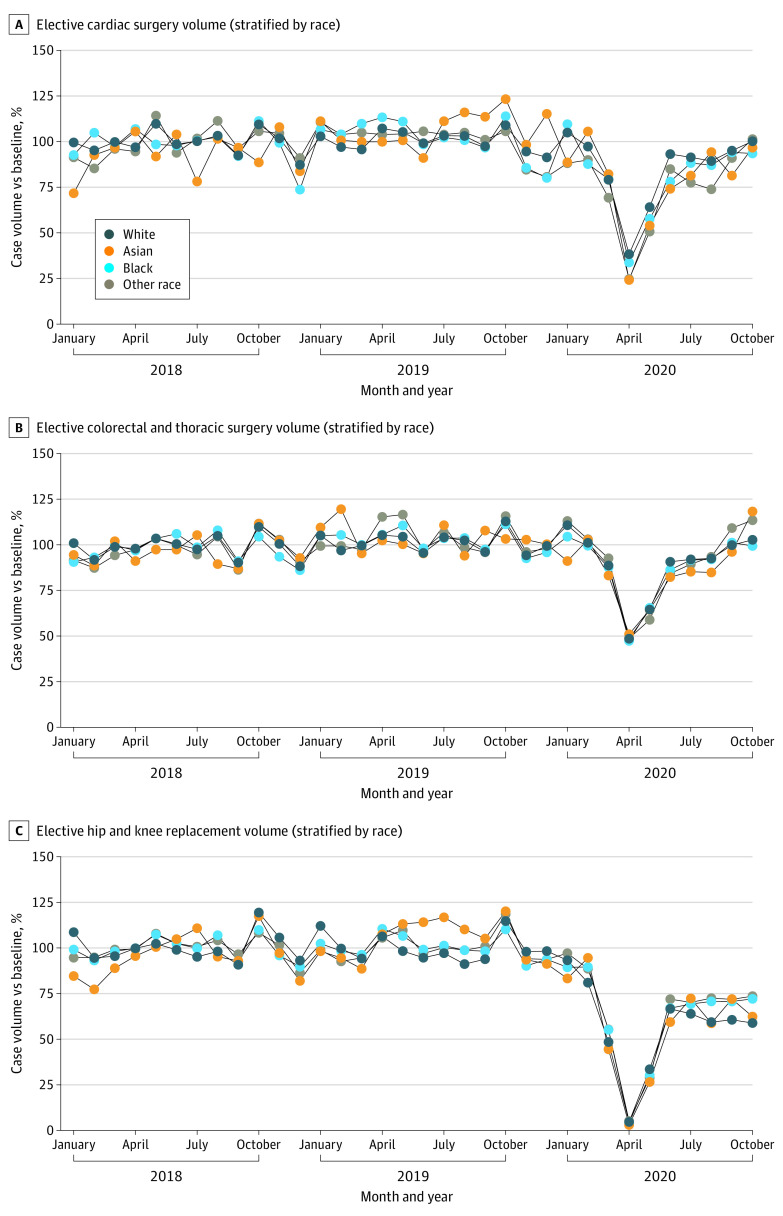
Elective Surgical Case Volumes for Cardiac Surgery, Colorectal and Thoracic Surgery, and Hip and Knee Replacements Stratified by Race Monthly case volumes were normalized by dividing monthly case volume by mean case volume during the baseline period (January 1, 2018, to February 29, 2020).

The results of the analyses examining the changes in elective surgical case volumes during the surge period for patients of Asian, Black, and other race undergoing cardiac or CABG surgery, thoracic or colorectal surgery, or orthopedic surgery are shown in Figure 4 and eFigure 4 in the Supplement. Unadjusted surgical case volumes for patients of Asian, Black, and other race undergoing cardiac or CABG surgery, thoracic or colorectal surgery, or orthopedic surgery did not decrease significantly more than for White patients undergoing the same operations during the surge period with the exception of individuals of other race undergoing cardiac surgery (IRR, 0.80; 95% CI, 0.73-0.89; *P* < .001). During the postsurge period, unadjusted surgical case volumes for patients of Asian, Black, and other race were not significantly different than for White patients with the exception of Black patients (unadjusted IRR, 1.13; 95% CI, 1.10-1.15; *P* < .001) and patients of other race (IRR, 1.12; 95% CI, 1.08-1.17; *P* < .001) undergoing hip and knee surgery, who exhibited increased surgical case volumes compared with White patients.

The results for the adjusted analyses are shown in Figure 3 and eFigure 4 and eTables 2 and 3 in the Supplement. These results indicate that patients of Black, Asian, and other race did not exhibit greater decreases in surgical case volumes after adjusting for age, sex, comorbidity count, and surgical procedure compared with White patients during the surge and postsurge periods.

## Discussion

Using national data on nearly 3.5 million patients undergoing major inpatient surgical procedures at 719 hospitals, this cross-sectional study found that the CMS’s moratorium on elective operations did not disproportionately reduce elective surgical case volumes for racial minority groups in the US during the first wave of the COVID-19 pandemic. Elective surgical case volumes decreased by 51% during the first wave of COVID-19 cases between March 1, 2020, and May 31, 2020. Individuals of Asian, Black, White, and other race experienced similar reductions in elective case volumes during the first wave of the pandemic. Elective case volumes rebounded during the recovery period between June and October 2020 to 85% of baseline. Elective surgery case volumes for individuals of Asian, Black, and other race recovered slightly more than for White individuals. We also found that Black and White individuals experienced similar reductions in the number of elective cardiac operations during the first wave and recovery. This finding is particularly important because, historically, Black individuals have less access to lifesaving invasive cardiac therapies,^10,16^ and COVID-19 is known to increase mortality up to 10-fold in patients with underlying cardiac disease.^17^

The decision to delay elective and nonessential operations to free physicians and nurses to care for patients during the first COVID-19 wave was critical in the CMS’s strategy to prevent the health care system from being overwhelmed.^6^ In the Veterans Administration health care system, the cancellation of elective operations freed 78% of surgical intensive care unit beds for patients with COVID-19.^8^ However, limiting access to surgical care could also lead to excess deaths from non–COVID-19 causes. For example, although the number of cardiac cases decreased by 52.7% overall during the first COVID-19 wave,^18^ the rebound in cardiac surgery that followed did not compensate for this reduction in case volumes.^18^ This attrition in cardiac operations is concerning because surgical delays for common cardiac operations are associated with increased mortality.^19,20^

Our study is the first, to our knowledge, to examine whether COVID-19 caused greater reductions in major surgery across the US during the first wave of the pandemic in Black and other racial minority individuals compared with White individuals. In 2020, the pandemic and the murder of George Floyd were a turning point in the public awareness of the structural barriers (such as residential and economic segregation, low-resource environments, unequal access to health care, and lack of health insurance) that prevent racial and ethnic minority groups from achieving the same health outcomes as their White counterparts.^21,22^ Before COVID-19, racial and ethnic minority individuals had shorter life expectancies^23^; were less likely to undergo major, high-cost surgical procedures,^10^ including CABG,^24^ arthroplasties,^25^ and renal transplants^26^; and were more likely to undergo limb amputation.^27^ COVID-19 has infected and killed greater numbers of people of minority race and ethnicity than White people because of the higher risk of exposure to COVID-19, greater comorbidity burden, and a higher likelihood of hospitalization of individuals of races other than White in underresourced safety-net hospitals.^22,28^ Our finding that COVID-19 did not worsen preexisting disparities in surgical care is somewhat surprising in light of the otherwise disproportionate effect of COVID-19 on the health of people of minority race and ethnicity. Nonetheless, it is reassuring that the US health care system did not respond to this unprecedented health care crisis by further limiting access to surgical care for racial minority individuals.

### Limitations

This analysis has several significant limitations. First, unlike national studies^10,29^ that examined disparities in the use of procedures in Medicare beneficiaries, we could not calculate utilization rates. Studies based on Medicare patients can identify the population of Medicare patients to calculate utilization rates using the denominator file. Our study, however, is based on all-payer data for a large cohort of academic and affiliated hospitals for which the population data necessary to calculate utilization rates are not available. Instead, we assumed that the size and racial composition of the patient populations served by the hospitals in our sample did not change between the baseline period and the first COVID-19 wave. Although possible, we believe that it is unlikely that our main finding—that the rationing of surgical care was not associated with disproportionately reduced surgical case volumes in racial minority individuals—was attributable to an increase in the number of racial minority persons in the regional health care markets served by the hospitals in our sample.

Second, our study only examines the association of COVID-19 with the relative decrease in the number of operations performed on racial minority individuals compared with White individuals. We did not examine the association of COVID-19 with disparities in health outcomes, such as mortality, complications, readmissions, and patient-reported outcomes. Further work is necessary to characterize the association of COVID-19 with disparities in surgical care and outcomes.

Third, our study is not population based and may not be generalizable to the rest of the US. Nonetheless, our study is the largest multicenter study to date. It is based on a large sample of US hospitals, including nearly all major academic medical centers, and is not limited to older Americans. Although it will be necessary to extend this study using national Medicare data, such a study will, by necessity, be limited to older Americans and not generalizable to the entire US population.

Fourth, our study, like other studies examining utilization and access to care,^10,29,30,31,32^ does not distinguish between appropriate and unnecessary procedures. Although it is possible to define appropriate and inappropriate use services for some discrete cases,^33,34^ this definition is challenging using administrative data for the wide range of surgical procedures included in our study even when population data are available.

Fifth, we could not examine the impact of COVID-19 on Hispanic individuals because of the large amount of missing data on ethnicity. Sixth, our study is limited to adult patients and does not examine the association of COVID-19 with operations performed in pediatric patients.

Seventh, our study did not examine the impact of COVID-19 on patients who were uninsured or had Medicaid insurance. Racial minority individuals without insurance may constitute one of the most vulnerable populations. We did not perform this analysis because the pandemic led to increases in uninsured people.^35^ Thus, decreases in surgical care among uninsured and Medicaid patients could be masked by increases in the numbers of individuals without insurance or with Medicaid insurance.

Eighth, our adjusted analysis controlled for a limited number of patient-level factors. However, although the unadjusted analysis showed that the CMS’s moratorium was not associated with greater reduction in surgical case volumes for minority individuals than White individuals, the adjusted analyses suggested that the moratorium was associated with less of a reduction for minority individuals than White individuals. In light of this finding, it is unlikely that more comprehensive adjustment would have demonstrated increasing disparities.

## Conclusions

The moratorium on nonessential operations led to a 51% decrease in elective surgical procedures in a national cohort of US hospitals. This first wave of COVID-19 infections was not associated with a greater reduction in elective surgical procedures in racial minority persons than White persons. These findings suggest that rationing of surgical care at the onset of the pandemic did not disproportionately affect racial minority persons. Our results suggest that, in a future pandemic, national efforts to limit elective surgery to increase hospital capacity may be implemented without increasing disparities in access to surgical care. More work is necessary to determine the association between COVID-19 and surgical outcomes and, in particular, whether racial minority patients experienced worse outcomes than White patients during the pandemic.

## Notes

Supplement. **eFigure 1.** Flow Diagram Describing Selection of Cases in the Analytic Cohort **eFigure 2.** Overall Surgical Case Volumes Stratified by Surgical Urgency **eFigure 3.** Changes in Surgical Case Volumes During the Surge and Post Periods Compared to the Baseline Period Stratified by Race and Surgical Urgency **eFigure 4.** Changes in Elective Surgical Case Volumes During the Surge and Post Periods Compared to Surgical Case Volumes for White Patients During the Baseline Period Stratified by Race and Surgical Procedure **eTable 1.** Hospital Characteristics **eTable 2.** Model 1 Coefficients **eTable 3.** Model 2 Coefficients
