# Supplementary material for: Association Between the COVID-19 Pandemic and Disparities in Access to Major Surgery in the US
Source: JAMA Netw Open. 2022 May 23;5(5):e2213527. doi: 10.1001/jamanetworkopen.2022.13527 (PMC9127559; doi:10.1001/jamanetworkopen.2022.13527)
Supplement: Supplement. — eFigure 1. Flow Diagram Describing Selection of Cases in the Analytic Cohort eFigure 2. Overall Surgical Case Volumes Stratified by Surgical Urgency eFigure 3. Changes in Surgical Case Volumes During the Surge and Post Periods Compared to the Baseline Period Stratified by Race and Surgical Urgency eFigure 4. Changes in Elective Surgical Case Volumes During the Surge and Post Periods Compared to Surgical Case Volumes for White Patients During the Baseline Period Stratified by Race and Surgical Procedure eTable 1. Hospital Characteristics eTable 2. Model 1 Coefficients eTable 3. Model 2 Coefficients [file jamanetwopen-e2213527-s001.pdf]

Supplementary Online Content

Glance LG, Chandrasekar EK, Shippey E, et al. Association between the COVID-19 pandemic and disparities in access to major surgery in the US. *JAMA Netw Open*. 2022;5(5):e2213527. doi:10.1001/jamanetworkopen.2022.13527

eFigure 1. Flow Diagram Describing Selection of Cases in the Analytic Cohort
eFigure 2. Overall Surgical Case Volumes Stratified by Surgical Urgency
eFigure 3. Changes in Surgical Case Volumes During the Surge and Post Periods Compared to the Baseline Period Stratified by Race and Surgical Urgency
eFigure 4. Changes in Elective Surgical Case Volumes During the Surge and Post Periods Compared to Surgical Case Volumes for White Patients During the Baseline Period Stratified by Race and Surgical Procedure
eTable 1. Hospital Characteristics
eTable 2. Model 1 Coefficients
eTable 3. Model 2 Coefficients

This supplementary material has been provided by the authors to give readers additional information about their work.

© 2022 Glance LG et al. *JAMA Network Open.*

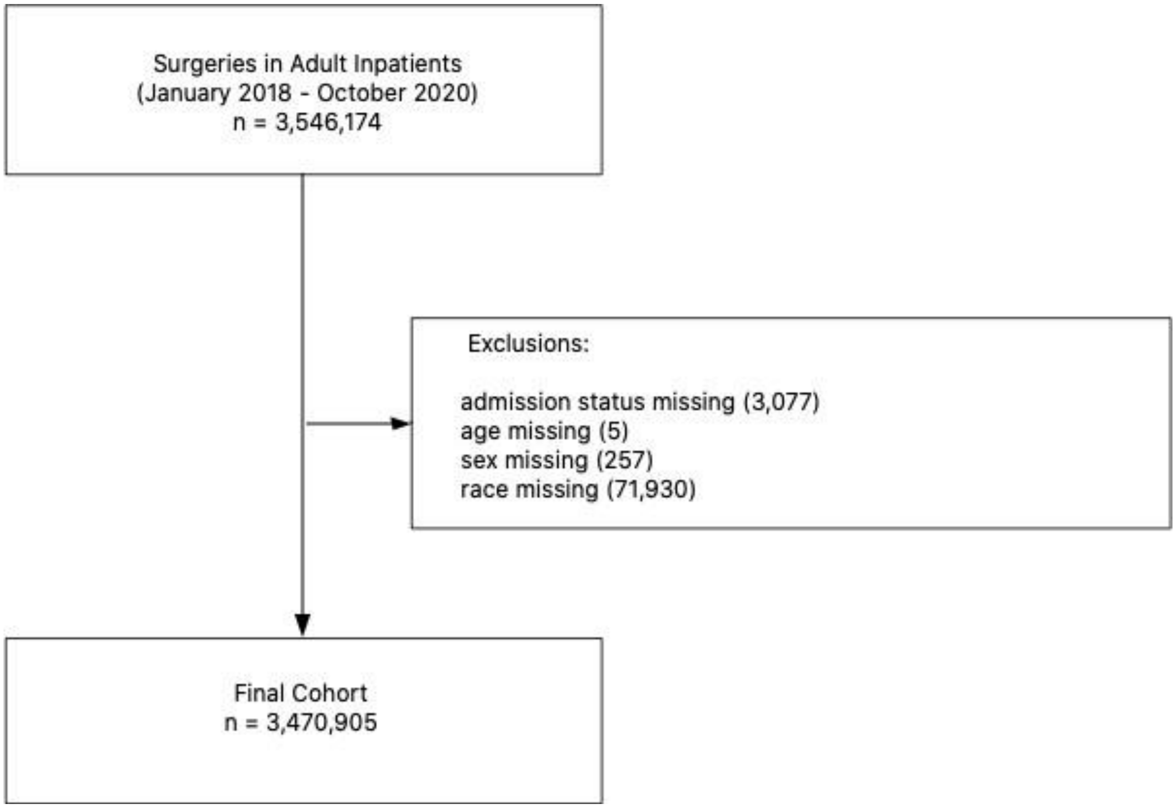

eFigure 1. Flow diagram describing selection of cases in the analytic cohort.

© 2022 Glance LG et al. *JAMA Network Open.*

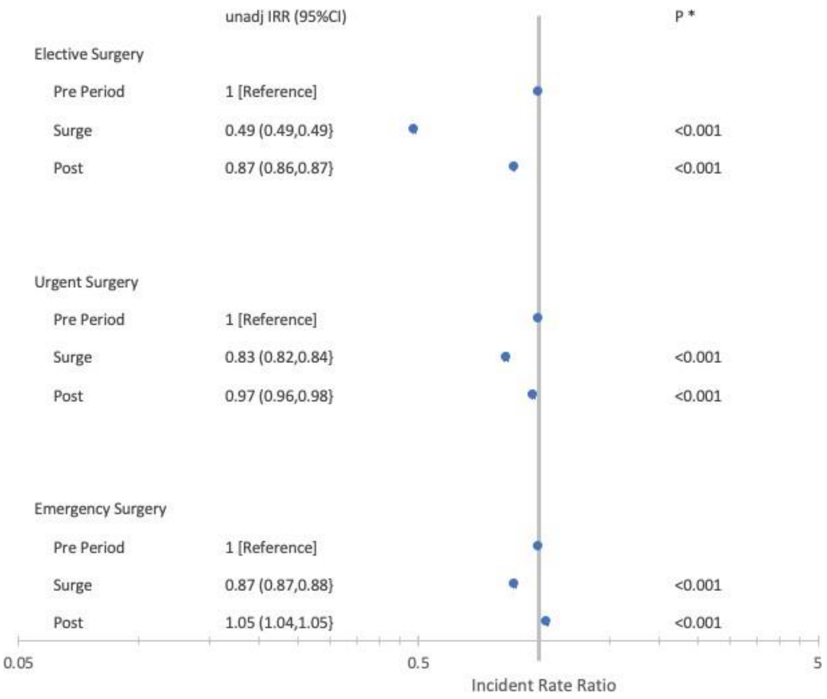

eFigure 2. Relative surgical case volumes stratified by surgical urgency for surge period (March 1, 2020 to May 31, 2020) and post period (June 1, 2020 to October 31, 2020) compared to baseline [pre] period (Jan 1, 2018 to February 29, 2020).
IRR – incident rate ratio (adjusted)
*adjusted P values

© 2022 Glance LG et al. *JAMA Network Open.*

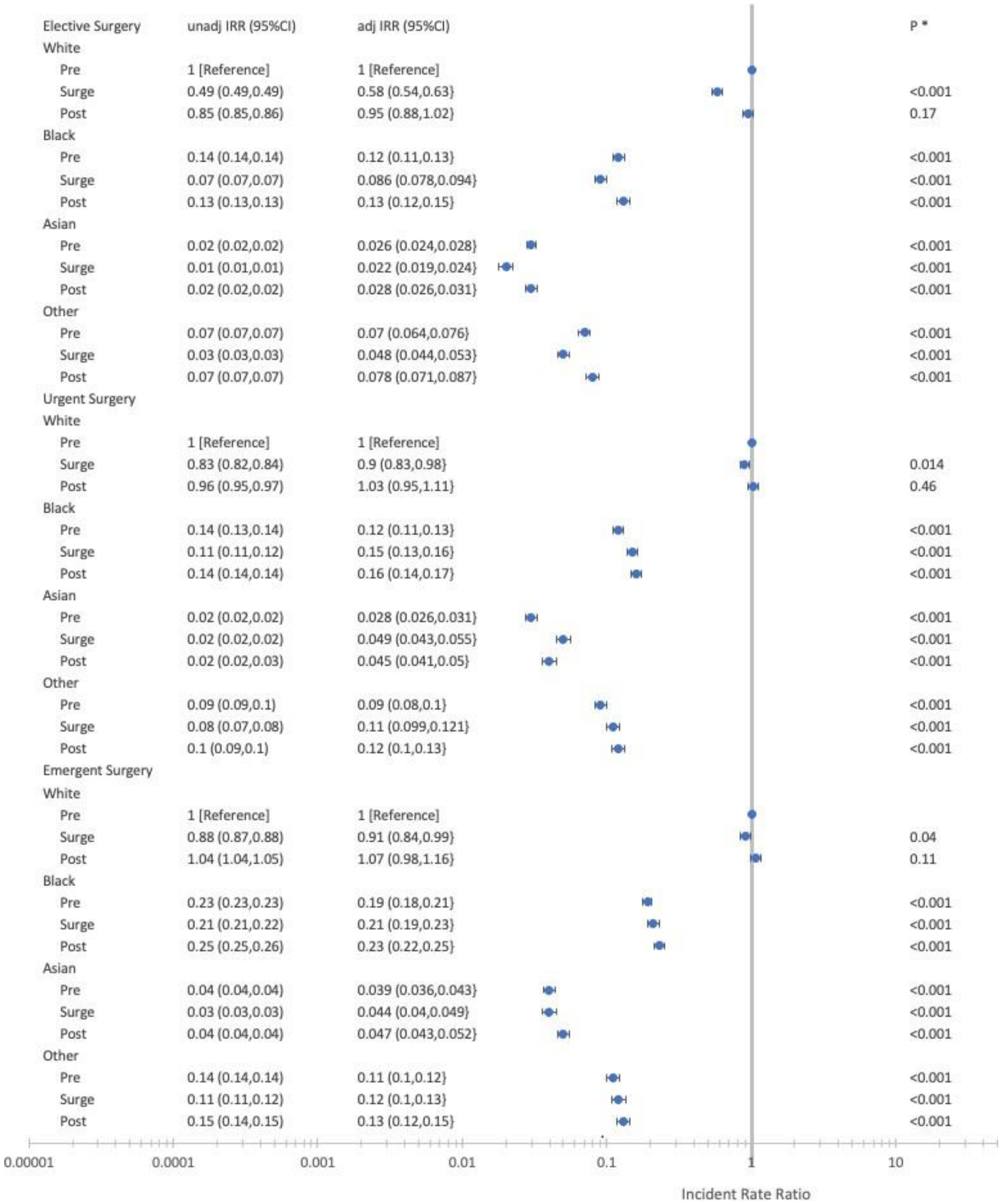

Elective Surgery	unadj IRR (95%CI)	adj IRR (95%CI)	P *
White			
Pre	1 [Reference]	1 [Reference]	
Surge	0.49 (0.49,0.49)	0.58 (0.54,0.63)	<0.001
Post	0.85 (0.85,0.86)	0.95 (0.88,1.02)	0.17
Black			
Pre	0.14 (0.14,0.14)	0.12 (0.11,0.13)	<0.001
Surge	0.07 (0.07,0.07)	0.086 (0.078,0.094)	<0.001
Post	0.13 (0.13,0.13)	0.13 (0.12,0.15)	<0.001
Asian			
Pre	0.02 (0.02,0.02)	0.026 (0.024,0.028)	<0.001
Surge	0.01 (0.01,0.01)	0.022 (0.019,0.024)	<0.001
Post	0.02 (0.02,0.02)	0.028 (0.026,0.031)	<0.001
Other			
Pre	0.07 (0.07,0.07)	0.07 (0.064,0.076)	<0.001
Surge	0.03 (0.03,0.03)	0.048 (0.044,0.053)	<0.001
Post	0.07 (0.07,0.07)	0.078 (0.071,0.087)	<0.001
Urgent Surgery			
White			
Pre	1 [Reference]	1 [Reference]	
Surge	0.83 (0.82,0.84)	0.9 (0.83,0.98)	0.014
Post	0.96 (0.95,0.97)	1.03 (0.95,1.11)	0.46
Black			
Pre	0.14 (0.13,0.14)	0.12 (0.11,0.13)	<0.001
Surge	0.11 (0.11,0.12)	0.15 (0.13,0.16)	<0.001
Post	0.14 (0.14,0.14)	0.16 (0.14,0.17)	<0.001
Asian			
Pre	0.02 (0.02,0.02)	0.028 (0.026,0.031)	<0.001
Surge	0.02 (0.02,0.02)	0.049 (0.043,0.055)	<0.001
Post	0.02 (0.02,0.03)	0.045 (0.041,0.05)	<0.001
Other			
Pre	0.09 (0.09,0.1)	0.09 (0.08,0.1)	<0.001
Surge	0.08 (0.07,0.08)	0.11 (0.099,0.121)	<0.001
Post	0.1 (0.09,0.1)	0.12 (0.1,0.13)	<0.001
Emergent Surgery			
White			
Pre	1 [Reference]	1 [Reference]	
Surge	0.88 (0.87,0.88)	0.91 (0.84,0.99)	0.04
Post	1.04 (1.04,1.05)	1.07 (0.98,1.16)	0.11
Black			
Pre	0.23 (0.23,0.23)	0.19 (0.18,0.21)	<0.001
Surge	0.21 (0.21,0.22)	0.21 (0.19,0.23)	<0.001
Post	0.25 (0.25,0.26)	0.23 (0.22,0.25)	<0.001
Asian			
Pre	0.04 (0.04,0.04)	0.039 (0.036,0.043)	<0.001
Surge	0.03 (0.03,0.03)	0.044 (0.04,0.049)	<0.001
Post	0.04 (0.04,0.04)	0.047 (0.043,0.052)	<0.001
Other			
Pre	0.14 (0.14,0.14)	0.11 (0.1,0.12)	<0.001
Surge	0.11 (0.11,0.12)	0.12 (0.1,0.13)	<0.001
Post	0.15 (0.14,0.15)	0.13 (0.12,0.15)	<0.001

eFigure 3. Relative changes in surgical case volumes during the Surge and Post periods compared to the baseline period (March 1, 2020 to May 31, 2020) stratified by race and surgical urgency (elective, urgent, emergent). Results are based on model 1 in which race and time period are combined into a single categorical variable.

IRR – incident rate ratio (adjusted)
*adjusted P values

© 2022 Glance LG et al. *JAMA Network Open.*

SURGE	unadj IRR (95%CI)	adj IRR (95%CI)	P *
Cardiac Surgery			
White	1 [Reference]	1 [Reference]	
Black	0.94 (0.87,1.03)	1.06 (0.79,1.42)	0.693
Asian	0.89 (0.77,1.02)	1.13 (0.86,1.48)	0.393
Other	0.8 (0.73,0.89)	0.88 (0.67,1.14)	0.319
Thoracic & Colorectal Surgery			
White	1 [Reference]	1 [Reference]	
Black	1 (0.95,1.06)	1.03 (0.78,1.36)	0.23
Asian	0.93 (0.84,1.04)	1.02 (0.67,1.34)	0.15
Other	0.99 (0.92,1.07)	1.16 (0.77,1.57)	0.97
Orthopedic Surgery			
White	1 [Reference]	1 [Reference]	
Black	1.04 (0.99,1.09)	1.1 (0.82,1.47)	0.534
Asian	0.85 (0.74,0.99)	0.9 (0.67,1.22)	0.501
Other	0.93 (0.86,1)	1.03 (0.77,1.37)	0.86
POST SURGE			
Cardiac Surgery			
White	1 [Reference]	1 [Reference]	
Black	0.94 (0.89,1)	1.02 (0.78,1.34)	0.886
Asian	0.91 (0.83,1)	0.96 (0.76,1.23)	0.767
Other	0.92 (0.87,0.98)	0.96 (0.74,1.23)	0.734
Thoracic & Colorectal Surgery			
White	1 [Reference]	1 [Reference]	
Black	0.99 (0.96,1.03)	0.98 (0.72,1.33)	0.892
Asian	0.95 (0.89,1.02)	0.98 (0.74,1.3)	0.901
Other	1.03 (0.98,1.08)	1.03 (0.76,1.4)	0.839
Orthopedic Surgery			
White	1 [Reference]	1 [Reference]	
Black	1.13 (1.1,1.15)	1.06 (0.79,1.42)	0.716
Asian	1.02 (0.95,1.1)	0.87 (0.65,1.18)	0.373
Other	1.12 (1.08,1.17)	1.08 (0.82,1.44)	0.57

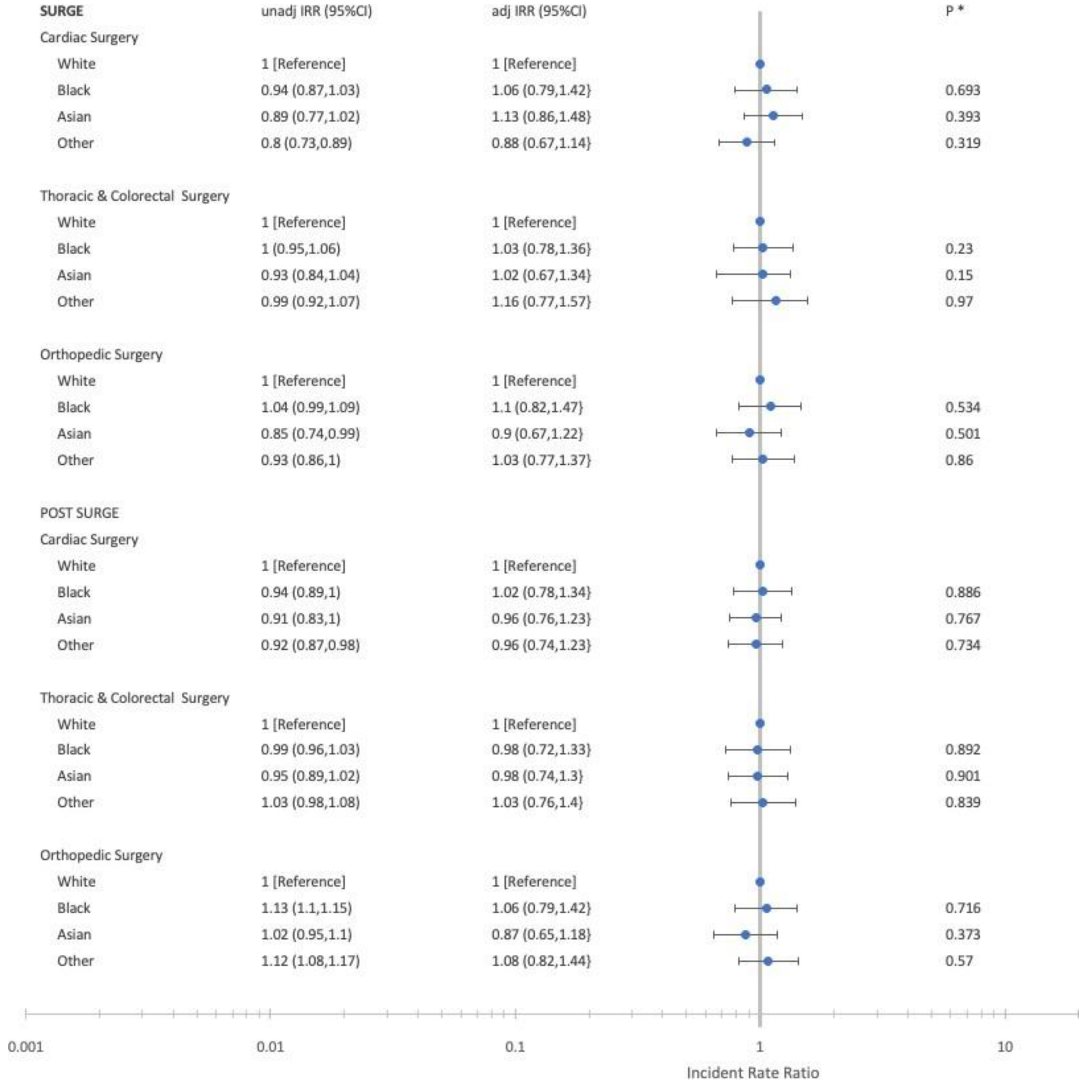

eFigure 4. Relative changes in elective surgical case volumes during the Surge and Post periods compared to surgical case volumes for White patients during the baseline period (March 1, 2020 to May 31, 2020) stratified by race and surgical procedure. Results are based on model 2 in which race and time period are specified as main effects.

IRR – incident rate ratio (adjusted)
*adjusted P values

© 2022 Glance LG et al. *JAMA Network Open.*

eTable 1. Hospital characteristics.

**Census zone**	
Midwest - East North Central	155 (21.6)
Midwest - West North Central	88 (12.2)
Northeast - Middle Atlantic	103 (14.3)
Notheast - New England	48 (6.7)
South - East South Central	19 (2.6)
South - South Atlantic	119 (16.6)
South - West South Central	62 (8.6)
West - Mountain	61 (8.5)
West - Pacific	57 (7.9)
missing	7 (1)
**Bedsize**	
<100	342 (47.6)
100-249	142 (19.8)
250-499	121 (16.8)
500 -	114 (15.9)
**Covid Burden During Surge, %**	
<= 5%	351 (48.8)
5.1-10%	129 (17.9)
10.1-25%	120 (16.7)
>25%	84 (11.7)
missing	35 (4.9)

© 2022 Glance LG et al. *JAMA Network Open.*

eTable 2. Model 1 coefficients.

	Elective				Urgent				Emergent			
	Unadjusted		Adjusted		Unadjusted		Adjusted		Unadjusted		Adjusted	
	IRR (95% CI)	P	IRR (95% CI)	P	IRR (95% CI)	P	IRR (95% CI)	P	IRR (95% CI)	P	IRR (95% CI)	P
Race												
White	reference				reference		reference		reference			
Black	0.14 (0.14,0.14)	P<.001	0.12 (0.11,0.13)	P<.001	0.14 (0.13,0.14)	P<.001	0.12 (0.11,0.13)	P<.001	0.23 (0.23,0.23)	P<.001	0.19 (0.18,0.21)	P<.001
Asian	0.02 (0.02,0.02)	P<.001	0.03 (0.02,0.03)	P<.001	0.02 (0.02,0.02)	P<.001	0.03 (0.03,0.03)	P<.001	0.04 (0.04,0.04)	P<.001	0.04 (0.04,0.04)	P<.001
Other race	0.07 (0.07,0.07)	P<.001	0.07 (0.06,0.08)	P<.001	0.09 (0.09,0.1)	P<.001	0.09 (0.08,0.1)	P<.001	0.14 (0.14,0.14)	P<.001	0.11 (0.1,0.12)	P<.001
Time Period				`								
Pre	reference		reference		reference		reference		reference		reference	
Surge	0.49 (0.49,0.49)	P<.001	0.58 (0.54,0.63)	P<.001	0.83 (0.82,0.84)	P<.001	0.9 (0.83,0.98)	0.014	0.88 (0.87,0.88)	P<.001	0.91 (0.84,0.99)	0.04
Post	0.85 (0.85,0.86)	P<.001	0.95 (0.88,1.02)	0.17	0.96 (0.95,0.97)	P<.001	1.03 (0.95,1.11)	0.46	1.04 (1.04,1.05)	P<.001	1.07 (0.98,1.16)	0.11
Time X Race												
Surge X White	reference				reference		reference		reference		reference	
Surge X Black	0.99 (0.97,1.01)	0.36	1.2 (1.06,1.37)	0.006	1.01 (0.97,1.06)	0.450	1.31 (1.15,1.5)	P<.001	1.04 (1.02,1.06)	P<.001	1.19 (1.05,1.34)	0.006
Surge X Asian	1.08 (1.03,1.14)	0.001	1.43 (1.25,1.64)	P<.001	0.96 (0.87,1.06)	0.45	1.9 (1.62,2.22)	P<.001	0.91 (0.87,0.95)	P<.001	1.25 (1.09,1.43)	0.001
Surge X Other race	0.97 (0.95,1)	0.05	1.19 (1.05,1.36)	0.009	1 (0.95,1.05)	0.96	1.32 (1.15,1.52)	P<.001	0.95 (0.93,0.98)	P<.001	1.14 (0.97,1.34)	0.11
Post X White	reference		reference		reference		reference		reference		reference	
Post X Black	1.08 (1.06,1.09)	P<.001	1.16 (1.01,1.32)	0.03	1.07 (1.03,1.1)	P<.001	1.24 (1.09,1.4)	0.001	1.05 (1.03,1.06)	P<.001	1.14 (1.01,1.28)	0.03
Post X Asian	1.04 (1.01,1.07)	0.007	1.15 (1.02,1.3)	0.03	1.05 (0.98,1.13)	0.16	1.54 (1.34,1.77)	P<.001	0.97 (0.94,1)	0.06	1.13 (1,1.28)	0.06
Post X Other race	1.1 (1.08,1.12)	P<.001	1.19 (1.03,1.36)	0.02	1.07 (1.03,1.11)	P<.001	1.22 (1.07,1.4)	0.004	1.02 (1,1.04)	0.03	1.11 (0.95,1.3)	0.18
Sex												
Male			reference				reference				reference	
Female			1.15 (1.11,1.19)	P<.001			0.99 (0.95,1.03)	0.69			0.93 (0.89,0.96)	P<.001
Age												
18-30			reference				reference				reference	
31-50			3.19 (2.83,3.59)	P<.001			2.32 (2.06,2.61)	P<.001			1.97 (1.74,2.22)	P<.001
51-64			4.62 (4.18,5.11)	P<.001			3.09 (2.78,3.43)	P<.001			2.37 (2.14,2.64)	P<.001

© 2022 Glance LG et al. *JAMA Network Open.*

65-74	3.88 (3.51,4.29)	P<.001	2.38 (2.14,2.65)	P<.001	1.69 (1.52,1.88)	P<.001
75-79	1.28 (1.15,1.42)	P<.001	0.9 (0.81,1.01)	0.07	0.65 (0.58,0.72)	P<.001
80-84	0.66 (0.59,0.73)	P<.001	0.61 (0.54,0.68)	P<.001	0.46 (0.41,0.51)	P<.001
85-89	0.26 (0.23,0.29)	P<.001	0.37 (0.33,0.42)	P<.001	0.31 (0.27,0.35)	P<.001
90 -	0.09 (0.08,0.1)	P<.001	0.23 (0.2,0.27)	P<.001	0.2 (0.18,0.23)	P<.001
Comorbidity count						
none	reference		reference		reference	
1-2	1.16 (1.1,1.21)	P<.001	1.69 (1.6,1.78)	P<.001	1.91 (1.82,2.01)	P<.001
3 or more	0.31 (0.29,0.33)	P<.001	1.03 (0.98,1.09)	0.23	1.35 (1.28,1.42)	P<.001
Procedure						
AAA repair	reference		reference		reference	
Amputation	17.6 (14.4,21.3)	P<.001	30 (24.8,36.3)	P<.001	53.1 (45.4,62.3)	P<.001
Appendectomy	5.5 (4.7,6.5)	P<.001	12.1 (9.8,15)	P<.001	45.4 (36.8,56.1)	P<.001
Bile duct, liver or pancreas	23.6 (20.3,27.3)	P<.001	10.4 (8.7,12.3)	P<.001	11.6 (10.1,13.4)	P<.001
Cardiac surgery	21.9 (18.9,25.3)	P<.001	21.4 (18.1,25.3)	P<.001	16.5 (14.4,18.9)	P<.001
CABG	14.9 (12.6,17.6)	P<.001	22.8 (19,27.4)	P<.001	16.1 (13.8,18.7)	P<.001
CABG with IMA (no vein)	1.6 (1.4,1.9)	P<.001	2.2 (1.8,2.7)	P<.001	1.5 (1.3,1.8)	P<.001
Carotid endarterectomy	11.8 (10,14)	P<.001	3.5 (2.9,4.2)	P<.001	5.1 (4.4,5.9)	P<.001
Cholecystectomy	7.8 (6.7,9.1)	P<.001	25.3 (21,30.6)	P<.001	79.9 (67.2,95)	P<.001
Colon surgery	40.3 (34.9,46.6)	P<.001	17.4 (14.7,20.5)	P<.001	37.6 (32.9,43.1)	P<.001
Craniotomy	30.9 (26.3,36.1)	P<.001	33.5 (28.4,39.4)	P<.001	71.2 (62.5,81.2)	P<.001
Spinal fusion	47.1 (40.6,54.6)	P<.001	14.3 (12.1,16.9)	P<.001	20.5 (17.9,23.5)	P<.001
Fracture surgery	10.3 (8.7,12.3)	P<.001	27.6 (22.8,33.5)	P<.001	106.4 (90.5,125)	P<.001
Gastric surgery	34.7 (28.2,42.6)	P<.001	8 (6.7,9.5)	P<.001	13.8 (12,15.8)	P<.001
Hip arthroplasty	47.7 (40.8,55.8)	P<.001	21.5 (17.6,26.2)	P<.001	60.4 (50.5,72.2)	P<.001
Knee arthroplasty	63.3 (54.2,74)	P<.001	9.1 (7.6,10.9)	P<.001	5.7 (4.9,6.6)	P<.001
Laminectomy	13.3 (11.5,15.4)	P<.001	7.4 (6.2,8.7)	P<.001	9.7 (8.4,11.1)	P<.001
Peripheral vascular bypass	10.9 (9.2,12.8)	P<.001	6 (5,7.2)	P<.001	7.2 (6.2,8.3)	P<.001
Small bowel surgery	22.4 (19,26.4)	P<.001	11.2 (9.5,13.3)	P<.001	24.9 (21.8,28.5)	P<.001
Thoracic surgery	26.8 (23.2,31.1)	P<.001	13.7 (11.6,16.3)	P<.001	19.4 (16.8,22.3)	P<.001
Exploratory laparotomy	25.7 (21.6,30.5)	P<.001	26.2 (21.4,32.2)	P<.001	38.5 (33.4,44.4)	P<.001

© 2022 Glance LG et al. *JAMA Network Open.*

eTable 3. Model 2 coefficients.

	Elective				Urgent				Emergency			
	Unadjusted		Adjusted		Unadjusted		Adjusted		Unadjusted		Adjusted	
	IRR (95% CI)	P	IRR (95% CI)	P	IRR (95% CI)	P	IRR (95% CI)	P	IRR (95% CI)	P	IRR (95% CI)	P
Race & Time Period												
White & Pre	reference		reference		reference		reference		reference		reference	
White & Surge	0.49 (0.49,0.49)	<0.001	0.58 (0.54,0.63)	<0.001	0.83 (0.82,0.84)	<0.001	0.9 (0.83,0.98)	0.01	0.88 (0.87,0.88)	<0.001	0.91 (0.84,0.99)	0.04
White & Post	0.85 (0.85,0.86)	<0.001	0.95 (0.88,1.02)	0.17	0.96 (0.95,0.97)	<0.001	1.03 (0.95,1.11)	0.46	1.04 (1.04,1.05)	<0.001	1.07 (0.98,1.16)	0.11
Black & Pre	0.14 (0.14,0.14)	<0.001	0.12 (0.11,0.13)	<0.001	0.14 (0.13,0.14)	<0.001	0.12 (0.11,0.13)	<0.001	0.23 (0.23,0.23)	<0.001	0.19 (0.18,0.21)	<0.001
Black & Surge	0.07 (0.07,0.07)	<0.001	0.09 (0.08,0.09)	<0.001	0.11 (0.11,0.12)	<0.001	0.15 (0.13,0.16)	<0.001	0.21 (0.21,0.22)	<0.001	0.21 (0.19,0.23)	<0.001
Black & Post	0.13 (0.13,0.13)	<0.001	0.13 (0.12,0.15)	<0.001	0.14 (0.14,0.14)	<0.001	0.16 (0.14,0.17)	<0.001	0.25 (0.25,0.26)	<0.001	0.23 (0.22,0.25)	<0.001
Asian & Pre	0.022 (0.022,0.022)	<0.001	0.026 (0.024,0.028)	<0.001	0.024 (0.023,0.025)	<0.001	0.028 (0.026,0.031)	<0.001	0.037 (0.036,0.037)	<0.001	0.039 (0.036,0.043)	<0.001
Asian & Surge	0.012 (0.011,0.012)	<0.001	0.022 (0.019,0.024)	<0.001	0.019 (0.018,0.021)	<0.001	0.049 (0.043,0.055)	<0.001	0.029 (0.028,0.03)	<0.001	0.044 (0.04,0.049)	<0.001
Asian & Post	0.019 (0.019,0.02)	<0.001	0.028 (0.026,0.031)	<0.001	0.024 (0.023,0.026)	<0.001	0.045 (0.041,0.05)	<0.001	0.037 (0.036,0.038)	<0.001	0.047 (0.043,0.052)	<0.001
Other race & Pre	0.071 (0.071,0.072)	<0.001	0.07 (0.064,0.076)	<0.001	0.09 (0.09,0.1)	<0.001	0.09 (0.08,0.1)	<0.001	0.14 (0.14,0.14)	<0.001	0.11 (0.1,0.12)	<0.001
Other race & Surge	0.034 (0.033,0.035)	<0.001	0.048 (0.044,0.053)	<0.001	0.08 (0.07,0.08)	<0.001	0.11 (0.1,0.12)	<0.001	0.11 (0.11,0.12)	<0.001	0.12 (0.1,0.13)	<0.001
Other race & Post	0.067 (0.066,0.068)	<0.001	0.078 (0.071,0.087)	<0.001	0.1 (0.09,0.1)	<0.001	0.12 (0.1,0.13)	<0.001	0.15 (0.14,0.15)	<0.001	0.13 (0.12,0.15)	<0.001
Sex												
Male			reference				reference				reference	
Female			1.15 (1.11,1.19)	<0.001			0.99 (0.95,1.03)	0.69			0.93 (0.89,0.96)	<0.001
Age												
18-30			reference				reference				reference	
31-50			3.19 (2.83,3.59)	<0.001			2.32 (2.06,2.61)	<0.001			1.97 (1.74,2.22)	<0.001
51-64			4.62 (4.18,5.11)	<0.001			3.09 (2.78,3.43)	<0.001			2.37 (2.14,2.64)	<0.001
65-74			3.88 (3.51,4.29)	<0.001			2.38 (2.14,2.65)	<0.001			1.69 (1.52,1.88)	<0.001
75-79			1.28 (1.15,1.42)	<0.001			0.9 (0.81,1.01)	0.07			0.65 (0.58,0.72)	<0.001
80-84			0.66 (0.59,0.73)	<0.001			0.61 (0.54,0.68)	<0.001			0.46 (0.41,0.51)	<0.001

© 2022 Glance LG et al. *JAMA Network Open.*

85-89	0.26 (0.23,0.29)	<0.001	0.37 (0.33,0.42)	<0.001	0.31 (0.27,0.35)	<0.001
90 -	0.09 (0.08,0.1)	<0.001	0.23 (0.2,0.27)	<0.001	0.2 (0.18,0.23)	<0.001
Comorbidity count						
none	reference		reference		reference	
1-2	1.16 (1.1,1.21)	<0.001	1.69 (1.6,1.78)	<0.001	1.91 (1.82,2.01)	<0.001
3 or more	0.31 (0.29,0.33)	<0.001	1.03 (0.98,1.09)	0.23	1.35 (1.28,1.42)	<0.001
Procedure						
AAA repair	reference		reference		reference	
Amputation	17.6 (14.4,21.3)	<0.001	30 (24.8,36.3)	<0.001	53.1 (45.4,62.3)	<0.001
Appendectomy	5.5 (4.7,6.5)	<0.001	12.1 (9.8,15)	<0.001	45.4 (36.8,56.1)	<0.001
Bile duct, liver or pancreas	23.6 (20.3,27.3)	<0.001	10.4 (8.7,12.3)	<0.001	11.6 (10.1,13.4)	<0.001
Cardiac surgery	21.9 (18.9,25.3)	<0.001	21.4 (18.1,25.3)	<0.001	16.5 (14.4,18.9)	<0.001
CABG	14.9 (12.6,17.6)	<0.001	22.8 (19,27.4)	<0.001	16.1 (13.8,18.7)	<0.001
CABG with IMA (no vein)	1.6 (1.4,1.9)	<0.001	2.2 (1.8,2.7)	<0.001	1.5 (1.3,1.8)	<0.001
Carotid endarterectomy	11.8 (10,14)	<0.001	3.5 (2.9,4.2)	<0.001	5.1 (4.4,5.9)	<0.001
Cholecystectomy	7.8 (6.7,9.1)	<0.001	25.3 (21,30.6)	<0.001	79.9 (67.2,95)	<0.001
Colon surgery	40.3 (34.9,46.6)	<0.001	17.4 (14.7,20.5)	<0.001	37.6 (32.9,43.1)	<0.001
Craniotomy	30.9 (26.3,36.1)	<0.001	33.5 (28.4,39.4)	<0.001	71.2 (62.5,81.2)	<0.001
Spinal fusion	47.1 (40.6,54.6)	<0.001	14.3 (12.1,16.9)	<0.001	20.5 (17.9,23.5)	<0.001
Fracture surgery	10.3 (8.7,12.3)	<0.001	27.6 (22.8,33.5)	<0.001	106.4 (90.5,125)	<0.001
Gastric surgery	34.7 (28.2,42.6)	<0.001	8 (6.7,9.5)	<0.001	13.8 (12,15.8)	<0.001
Hip arthroplasty	47.7 (40.8,55.8)	<0.001	21.5 (17.6,26.2)	<0.001	60.4 (50.5,72.2)	<0.001
Knee arthroplasty	63.3 (54.2,74)	<0.001	9.1 (7.6,10.9)	<0.001	5.7 (4.9,6.6)	<0.001
Laminectomy	13.3 (11.5,15.4)	<0.001	7.4 (6.2,8.7)	<0.001	9.7 (8.4,11.1)	<0.001
Peripheral vascular bypass	10.9 (9.2,12.8)	<0.001	6 (5,7.2)	<0.001	7.2 (6.2,8.3)	<0.001
Small bowel surgery	22.4 (19,26.4)	<0.001	11.2 (9.5,13.3)	<0.001	24.9 (21.8,28.5)	<0.001
Thoracic surgery	26.8 (23.2,31.1)	<0.001	13.7 (11.6,16.3)	<0.001	19.4 (16.8,22.3)	<0.001
Exploratory laparotomy	25.7 (21.6,30.5)	<0.001	26.2 (21.4,32.2)	<0.001	38.5 (33.4,44.4)	<0.001

© 2022 Glance LG et al. *JAMA Network Open.*
